# Recovery from Lightning Stroke

**Published:** 1875-09

**Authors:** 


Recovery from Lightning-Stroke.—In his valuable work on "The Maintenance of Health," Dr. Fothergill has the following on resuscitation after lightning-stroke: "Persons struck by lightning are not always dead when they appear to be so. There are few recoveries from this state, because no means are tried to restore the sufferer. In the tropics there are many instances of persons struck down by lightning, recovering after a heavy thunder shower; and it would appear that cold affusion to the body has a decided action in such cases. The injured cannot be harmed by the free use of cold water, and if only an occasional recovery took place it would be well worth the pains bestowed. The persons so injured should have cold water poured or dashed freely over them."—*From Popular Science Monthly for September.*

